# Basal cell carcinoma in farmers: an occupation group at high risk

**DOI:** 10.1007/s00420-015-1088-0

**Published:** 2015-10-13

**Authors:** Mateusz Szewczyk, Jakub Pazdrowski, Paweł Golusiński, Aleksandra Dańczak-Pazdrowska, Łukasz Łuczewski, Sławomir Marszałek, Ewa Majchrzak, Wojciech Golusiński

**Affiliations:** Department of Head and Neck Surgery, Greater Poland Cancer Centre, Poznan University of Medical Sciences, Garbary 15, 61-866 Poznan, Poland; Department of Biology and Environmental Studies, Poznan University of Medical Sciences, Dluga 1⁄2, 61-848 Poznan, Poland; Department of Dermatology, Poznan University of Medical Sciences, Przybyszewskiego 49, Poznan, Poland; Department of Rehabilitation in Internal Medicine, University School of Physical Education, Poznan, Poland

**Keywords:** Basal cell carcinoma, Farmers, Sun, Exposure, Recurrence, Occupation

## Abstract

**Introduction:**

Skin cancer is the most commonly diagnosed cancer type worldwide, and 80 % of skin cancers are basal cell carcinoma (BCC). The main risk factor for developing BCC is exposure to ultraviolet radiation (UVR), particularly high-dose exposure at a young age. Outdoor workers, particularly farmers, are at high risk of developing BCC. However, studies of BCC in this population are scant.

**Objective:**

To comprehensively evaluate all cases of BCC of the head and neck region treated during the years 2007–2013 at our hospital in Poland, and to compare the tumour characteristics in farmers to non-farmers.

**Materials and methods:**

Retrospective analysis of 312 patients treated for head and neck BCC during the study period (2007–2013).

**Results:**

Most patients (198 cases; 63 %) were males, with 114 females (37 %). Median age was 73 years (range 32–96 years). The most common tumour location was the nose and cheek (114 pts; 37 %) followed by the auricle (82 pts; 26 %), lips (54 pts; 18 %), scalp (26 pts; 8 %), and eye (36 pts; 12 %). The most common disease stage on presentation was stage T2 (104 pts, 33 %), followed by stage T1 (79 pts; 25 %), stage T3 (89 pts; 28 %), and stage T4 (40 pts; 14 %). By occupation, farmers accounted for 33 % of all patients (102 of 312 pts). The most common tumour localisations in the farmer subgroup were the nose and cheek (50 pts; 49 %; *p* < 0.001; odds ratio [OR] 2.19; 95 % confidence interval [CI] 1.35–3.57), followed by the auricle (32 pts; 31 %), scalp (16 pts; 16 %), ocular region (3 pts; 3 %), and lips (1 pt; 1 %). Patients in the farmer group were significantly younger than non-farmers (62 vs. 73 years; *p* < 0.001; OR 0.90, 95 % CI 0.88–0.93). Farmers were significantly more likely to present disease recurrence (27 vs. 12 % of cases; *p* < 0.001; OR 5.94; 95 % CI 2.86–12.33).

**Conclusion:**

The results highlight the increased incidence and risk of recurrence of BCC in farmers. It is therefore necessary to consider enhancing educational programmes and other preventative measures in this occupational group and to evaluate the effectiveness of such programmes.

## Introduction

Skin cancer is the most commonly diagnosed cancer type worldwide, and basal cell carcinoma (BCC) accounts for 80 % of all cases (Baxter et al. 2012). Although BCC is a malignant cancer, it very rarely metastasizes (<0.1 %). At diagnosis, most patients are in their sixth and seventh decade of life and men are twice as likely to develop BCC. The incidence of BCC has tripled in the last 30 years (Staples et al. 2006) and, in recent years, the incidence rate has increased significantly in younger patients and in females (De Vries et al. 2004; Sobjanek et al. 2012) due to the increasing use of artificial sources of ultraviolet radiation (UVR), primarily solar beds (Werher et al. 2012).

The main causative risk factor for BCC development is exposure to UVR (Lesiak et al. 2011; Wódz-Naskiewicz et al. 2011), which initiates carcinogenesis in skin basal cells through a destructive effect on cell DNA and immunosuppression (Osmola-Mańkowska et al. 2012). In Europe, incidence rates are lowest in Finland (Hannuksela et al. 1999) due to the decreased solar UVR exposure in that country. In Poland, BCC incidence is 13.3 male and 10.3 female cases per 100.000 (Wojciechowska et al. 2011). Histologically, five types of BCC have been described, with the most common being nodular BCC, which is associated with chronic exposure to UVR (Bastiaens et al. 1998). The time of first UVR exposure is very significant, and patients with high UVR exposure at <age 20 have a higher likelihood of developing BCC later in life (Walther et al. 2004). Other important risk factors include exposure to pesticides, ionising radiation, RTG radiation, and skin burns.

Given the primary role of early and long-term sun exposure in BCC, outdoor workers—particularly farmers—are at much greater risk of developing BCC. Indeed, outdoor workers are 43 % more likely to develop BCC (Bauer et al. 2011). However, as one recent review reported (Kearney et al. 2014), studies on farm workers, sun safety behaviour, and skin cancer are scant. For this reason, we carried out the present retrospective study, in which we comprehensively evaluate all cases of BCC of the head and neck region treated during the years 2007–2013 at our hospital in Poland, and we compare farmers to non-farmers to determine differences between the groups.

## Materials and methods

Retrospective analysis of 312 patients (pts) diagnosed and treated for head and neck BCC at the Head and Neck Department at the Greater Poland Cancer Centre (GPCC) of the University of Medical Sciences (Poznan, Poland) between 2007 and 2013. For comparison, patients were grouped into farmers (102 pts; 33 %) and non-farmers (210 pts; 67 %).

The statistical analysis was performed on Statistica 9.1 statistical software (StatSoft). The results were considered significant at *p* < 0.05. To assess for statistically significant differences between farmers and non-farmers, Shapiro–Wilk and student’s *t* test were used. Odds ratio was calculated with Chi-square test using the approximation of Woolf.

## Results

Out of 312 patients, 198 were males (63 %) and 114 females (37 %). Median age was 73 years (range 32–96 years). The most common tumour location (114 pts; 37 %) was the nose and cheek, followed by the auricle (82 pts; 26 %), lips (54 pts; 18 %), scalp (26 pts; 8 %), and eye (36 pts; 12 %). In the patient sample, no cases of multiple BCC were recorded on the day of the first admission to hospital.

The most common disease stage on presentation was stage T2 (104 pts; 33 %), followed by stage T1 (79 pts; 25 %), stage T3 (89 pts; 28 %), and stage T4 (40 pts; 14 %). None of the patients presented with metastases to regional lymph nodes. By occupation, farmers accounted for one-third of all patients in this sample (102 of 312 pts; 33 %). The most common tumour localisations in the farmer subgroup were the nose and cheek (50 pts; 49 %, *p* < 0.001; odds ratio [OR] 2.19; 95 % [CI] 1.35–3.57; in reference to nose and cheek localisation within non-farmers), followed by the auricle (32 pts; 31 %), scalp (16 pts; 16 %), ocular region (3 pts; 3 %) and lips (1 pt; 1 %). The average age of patients in the farmer group was 62 years versus 73 in non-farmers (*p* < 0.001; OR 0.90, 95 % CI 0.88–0.93) (Tables 1, 2). Of the 102 farmers, 36 (35 %) reported a family history of BCC. Head and neck recurrence was observed in 27 of the 102 farmers (27 %) versus 12 cases (12 %; *p* < 0.001; OR 5.94; 95 % CI 2.86–12.33) in non-farmers (Table 3). The most common BCC type in both groups was nodular BCC: farmers (58 cases; 57 %) and non-farmers (117 cases; 56 %). At admission, 78 of the 102 farmers (76 %) reported high-dose sun exposure before age 20 (>10 h/day outdoors), while the remaining 24 pts (24 %) reported average sun exposure before age 20 (between 7 and 10 h/day outdoors).

**Table 1:** Site distribution of BCC by occupation: farmers versus non-farmers

	Nose and cheek	Auricle	Lip	Eyelids	Scalp	Overall
Patients overall	114 (37 %)	82 (26 %)	54 (18 %)	36 (12 %)	26 (8 %)	312 (100 %)
Farmers	50 (49 %)	32 (31 %)	1 (1 %)	3 (3 %)	16 (16 %)	102 (100 %)
Non-farmers	64 (30 %)	50 (24 %)	53 (25 %)	33 (16 %)	10 (5 %)	210 (100 %)

**Table 2:** Age and sex distribution of BCC in the farmer and non-farmer groups

	Nose and cheek	Auricle	Lip	Eyelids	Scalp	Overall (100 % of patients)
Age 25–49 (F)	2 (29 %)	0	1 (14 %)	1 (14 %)	3 (43 %)	7
Age 50–65 (F)	17 (37 %)	21 (46 %)	0	2 (4 %)	6 (13 %)	46
Age 65–80 (F)	24 (67 %)	7 (19 %)	0	0	5 (14 %)	36
Age > 80 (F)	7 (54 %)	4 (31 %)	0	0	2 (15 %)	13
Total	50 (49 %)	32 (31 %)	1 (1 %)	3 (3 %)	16 (16 %)	102
Age 25–49 (NF)	2 (40 %)	1 (20 %)	2 (40 %)	0	0	5
Age 50–65 (NF)	11 (39 %)	7 (25 %)	6 (21 %)	3 (11 %)	1 (4 %)	28
Age 65–80 (NF)	43 (32 %)	34 (25 %)	36 (26 %)	18 (13 %)	5 (4 %)	136
Age > 80 (NF)	8 (20 %)	8 (20 %)	9 (22 %)	12 (29 %)	4 (9 %)	41
Total	64 (30 %)	50 (24 %)	53 (25 %)	33 (16 %)	10 (5 %)	210
Women (F)	21 (62 %)	9 (26 %)	0	1 (3 %)	3 (9 %)	34
Men (F)	29 (43 %)	23 (34 %)	1 (1 %)	2 (3 %)	13 (19 %)	68
Total	50 (49 %)	32 (31 %)	1 (1 %)	3 (3 %)	16 (16 %)	102
Women (NF)	22 (27 %)	24 (30 %)	18 (22 %)	13 (16 %)	3 (5 %)	80
Men (NF)	42 (32 %)	26 (20 %)	35 (27 %)	20 (15 %)	7 (6 %)	130
Total	64 (30 %)	50 (24 %)	53 (25 %)	33 (16 %)	10 (5 %)	210

*F* indicates farmer, *NF* non-farmer

**Table 3:** BCC recurrence by occupation

Location	Nose and cheek	Auricle	Lips	Eyelids	Scalp	Overall
Cases: farmers	50 (100 %)	32 (100 %)	1 (100 %)	3 (100 %)	16 (100 %)	102
Recurrences: farmers	17 (34 %)	6 (19 %)	0	1 (33 %)	3 (18 %)	27
Case: non-farmers	64 (100 %)	50 (100 %)	53 (100 %)	33 (100 %)	10 (100 %)	210
Recurrences: non-farmers	6 (9 %)	4 (8 %)	0	1 (3 %)	1 (10 %)	12

## Discussion

UVR is the strongest risk factor for BCC, as this study confirms. Farmers made up a disproportionately large percentage (33 %) of patients treated at our hospital for BCC during the study period. Moreover, farmers were significantly younger than non-farmers (62 vs. 73 years) at diagnosis and much more likely than non-farmers to report significant exposure to UVR before age 20. In addition, the risk of recurrence was significantly greater in farmers. These findings demonstrate that farmers are at greater risk of developing BCC, thus emphasising the need for better educational interventions to minimise the risks of developing BCC in this occupational group. The results of this current study are consistent with the published literature showing that outdoor workers are at greater risk of developing BCC (Mosterd et al. 2008), particularly in the head and neck region (Maia et al. 1995).

Despite the fact that one in three BCC patients was a farmer, the total count of farmers has likely been underestimated in this study. As other authors have reported, this underrepresentation is likely due, at least partially, to these patients’ unwillingness to be admitted to the hospital, a phenomenon that is especially notable in people with low social and educational status. As in other studies, such as Dogan (2007) and Radespiel-Tröger et al. (2009), we found that most of the farmers were males (male-to-female ratio 3:1). This finding is not unusual, as most outdoor work in the countryside is performed by males. However, this male–female difference may be partially attributable to other factors. Laboratory research carried out by Thomas–Ahner et al. (2007) in mice showed that male mice that received the same UVB radiation dose as female mice were more likely to develop BCC, and those males that developed BCC did so at an earlier age; in addition, they found that the disease itself was much more aggressive in the males. In our study, we found differences between sexes in terms of tumour localisation. Tumours of the scalp were more common in male farmers, although this may be attributable to hair loss in men, and thus hair may have been a protective factor in women.

The most common tumour location in both groups was the nose and cheek area. However, this site was significantly more common in farmers (nearly half of all cases) than in non-farmers (only 30 % of cases). Farmers were also more likely to present BCC of the scalp versus non-farmers (16 vs. 5 %). These findings are all consistent with the carcinogenic influence of UVR in the development of head and neck BCC, particularly in locations with high sun exposure. However, we also found that lip cancer accounted for 25 % of cases in the non-farmer group, whereas the incidence in farmers was negligible (only 1 %). Similarly, BCC of the eyelid was much more common in non-farmers (16 vs. 3 %). While the differences between farmers and non-farmers in certain tumour locations (i.e. scalp, nose & cheek) can be explained by the intense sun exposure in farmers, other differences in location (e.g. eyelid, lips) are harder to explain. Moreover, to our knowledge, little to no data have been published in the literature.

### Family history of BCC

More than one-third of patients in the farmer group reported a family history of BCC. In the non-farmer group, only 15 % reported a family history. Clearly, this difference can partially be explained by occupational risk, as family members of farmers are more likely to also be farmers. However, of all skin cancers, BCC has the largest familial association. In a study of 166 patients with head and neck BCC compared to a large (158 subjects) control group, Corona et al. (2001) found that sun overdosing in childhood and adolescence and a positive family history of BCC were the strongest independent risk factors for BCC development.

### Risk of recurrence

BCC has a high risk of recurrence (ranging from 5–25 %) after treatment. To enhance the potential to achieve curative radical dissection, optimal patient management requires close interdisciplinary cooperation between head and neck surgeons, dermatologists, plastic surgeons, pathologists, oncologists, and radiologists. Preoperative ultrasound examination of the tumour to measure the size (especially the depth of infiltration) with the use of a 20-MHz frequency linear transducer can significantly reduce the risk of relapse because it provides valuable information that helps to improve the surgical procedure (Danczak-Pazdrowska et al. 2012). In our study, we found that farmers had a significantly higher postoperative recurrence rate than non-farmers (27 vs. 6 %), although the reasons for this difference are not clear.

### Prevention

Epidemiological research proves the importance of education and other preventative measures to prevent BCC. For instance, an educational programme called Slip!Slop!Slap! (i.e. slip on a shirt, slop on some sun cream, and slap on a hat, respectively) was implemented in Australia in the 1980s. According to the results of a long-term follow-up study published in 2006, this programme helped to stop the increase in the incidence of skin cancer in that region (Staples et al. 2006). Prevention of both primary and recurrent tumours has obvious health benefits for the individuals involved, but such measures would help to save significant amounts of money: €3141/patient, according to Mosterd et al. (2008). In the USA, skin cancer is estimated to cost up to $426 million USD in treatments and lost productivity. In Germany, BCC has not yet been added to the official list of occupational diseases, but several authors have proposed doing so (Diepgen et al. 2012; Diepgen and Drexler 2004). In Poland, although no specific programme has been developed to combat skin cancer in the farmer population, efforts have been made to expand the National Cancer Combat Programme entitled “Improvement of the cancer data collection and registration system” to evaluate the level of public knowledge of health-promoting behaviours and to raise public awareness of cancer by providing informational materials and organising media campaigns to promote a healthy lifestyle (Dyzmann-Sroka and Malicki 2014). In 2005, the National Programme against Cancer Diseases (NPACD) was implemented to provide substantial funds for investment in new equipment, to develop screening and prevention programmes to improve cancer registries, and to carry out epidemiological studies (Malicki and Golusinski 2014). However, it is clear that more targeted programmes are needed for high-risk groups such as farm workers.

### Study limitations

Due to the retrospective nature of this study, we were not able to evaluate patients for UVR-induced skin damage. For the same reason, we do not have detailed data on the outdoor activities of the patient.

## Conclusion

Although farmers make up only 12.9 % of the population in Poland, in this study we have found that this occupational group accounts for 33 % of the group of patients who develop BCC. In addition, our data show that farmers develop BCC at a younger age and are more likely to suffer a recurrence. This adds useful information to the scarce data in the published literature and underscores the need to implement educational programmes and other preventative measures to prevent skin cancer among this high-risk group.

## References

[CR1] Bastiaens MT, Hoefnagel JJ, Bruijn JA, Westendorp RGJ, Vermeer BJ, Bouwes Bavinck JN (1998). Differences in age, site, distribution and sex between nodular and superficial basal cell carcinomas indicate different types of tumors. J Invest Dermatol.

[CR2] Bauer A, Diepgen TL, Schmitt J (2011). Is occupational solar ultraviolet irradiation a relevant risk factor for basal cell carcinoma?. A systematic review and meta-analysis of the epidemiological literature. Br J Dermatol.

[CR3] Baxter JM, Patel AN, Varma S (2012). Facial basal cell carcinoma. BMJ.

[CR5] Corona R, Dogliotti E, D’Errico M, Sera F, Iavarone I (2001). Risk factors for basal cell carcinoma in a mediterranean population: role of recreational sun exposure early in life. Arch Dermatol.

[CR6] Dańczak-Pazdrowska A, Polańska A, Silny W, Sadowska A, Osmola-Mańkowska A, Czarnecka-Operacz M (2012). Seemingly healthy skin in atopic dermatitis: observations with the use of high-frequency ultrasonography, preliminary study. Skin Res Technol.

[CR7] De Vries E, Louwman M, Bastiens M, de Gruijl F, Coebergh JW (2004). Rapid and continuous increases in incidence rates of basal cell carcinoma in the southwest Netherlands since 1973. J Invest Dermatol.

[CR8] Diepgen TL, Drexler H (2004). Skin cancer and occupational disease. Hautarzt.

[CR9] Diepgen TL, Fartasch M, Drexler H, Schmitt J (2012). Occupational skin cancer induced by ultraviolet radiation and its prevention. Br J Dermatol.

[CR10] Dogan G (2007). Basal cell carcinoma in outdoor versus indoor workers in Turkey. Int J Dermatol.

[CR11] Dyzmann-Sroka A, Malicki J (2014). Cancer incidence and mortality in the Greater Poland Region-Analysis of the year 2010 and future trends. Rep Pract Oncol Radiother.

[CR12] Hannuksela A, Pukkala E, Karvonen J (1999). Basal cell skin carcinoma and other nonmelanoma skin cancers in Finland from 1956 through 1995. Arch Dermatol.

[CR13] Kearney GD, Xu X, Balanay JA, Becker AJ (2014). Sun safety among farmers and farmworkers: a review. J Agromedicine.

[CR14] Lesiak A, Wódz-Naskiewicz K, Pawliczak R, Rogowski-Tylman M, Sysa-Jędrzejowska A (2011). The influence of vitamin D receptor gene polymorphism on basal cel carcinoma development in Polish population. Post Dermatol Alergol.

[CR15] Maia M, Proenca NG, de Morales JC (1995). Risk factors for basal cell carcinoma: a case control study. Rev Saude Publica.

[CR16] Malicki J, Golusinski W (2014). Challenges in organizing effective oncology service: inter-European variability in the example of head and neck cancers. Eur Arch Otorhinolaryngol.

[CR17] Mosterd K, Krekels GAM, Ostertag JU, Essers AB, Dirksen CD, Steijlen PM (2008). Surgical excision versus Mohs’ micrographic surgery for primary and recurrent basal-cell carcinoma of the face: a prospective randomised controlled trial with 5-years’ follow-up. Lancet Oncol.

[CR18] Osmola-Mańkowska A, Silny W, Dańczak-Pazdrowska A, Olek-Hrab K, Mańkowski B, Osmola K (2012). The sun–our friend or foe?. Ann Agric Environ Med.

[CR19] Radespiel-Tröger M, Meyer M, Pfahlberg A, Lausen B, Uter W, Gefeller O (2009). Outdoor work and skin cancer incidence: a registry-based study in Bavaria. Int Arch Occup Environ Health.

[CR20] Sobjanek M, Michajłowski J, Malek M, Zabłotna M, Włodarkiewicz A, Nowicki R (2012). Skin cancer in the elderly—epidemiological, clinical and surgical treatment analysis of 254 patients. Post Dermatol Alergol.

[CR21] Staples MP, Elwood M, Burton RC, Williams JL, Marks R, Giles GG (2006). Non-melanoma skin cancer in Australia: the 2002 national survey and trends since 1985. Med J Aust.

[CR22] Thomas-Ahner JM, Wulff BC, Tober KL (2007). Gender differences in UVB—induced skin carcinogenesis, inflammation and DNA damage. Cancer Res.

[CR23] Walther U, Kron M, Sander S, Sebastian G, Sander R, Peter RU (2004). Risk and protective factors for sporadic basal cell carcinoma: results of a two-centre case-control study in southern Germany. Clinical actinic elastosis may be a protective factor. Br J Dermatol.

[CR24] Werher MR, Shive M, Chren MM, Han J, Qureshi A, Linos E (2012). Indoor tanning and non-melanoma skin cancer: systematic review and meta—analysis. BMJ.

[CR25] Wódz-Naskiewicz K, Narbutt J, Rogowski-Tylman M, Pawliczak R, Sobjanek M, Sysa-Jędrzejowska A (2011). Association of methylenetetrahydrofolate reductase gene polymorphisms with basal cel carcinoma development. Post Dermatol Alergol.

[CR26] Wojciechowska U, Didkowska J, Zatoński W, Nowotwory złośliwe w Polsce w (2011) Published within the framework of the task, Cancer Registration” by the National Cancer Control Programme. Polish

